# First Evidence of Intracellular Bacteria *Cardinium* in Thermophilic Mite *Microzetorchestes emeryi* (Acari: Oribatida): Molecular Screening of Bacterial Endosymbiont Species

**DOI:** 10.1007/s00284-019-01717-5

**Published:** 2019-06-18

**Authors:** Edyta Konecka, Ziemowit Olszanowski

**Affiliations:** 1 Department of Microbiology, Faculty of Biology, Adam Mickiewicz University in Poznań, Umultowska 89, 61-614 Poznan, Poland; 2 Department of Animal Taxonomy and Ecology, Faculty of Biology, Adam Mickiewicz University in Poznań, Umultowska 89, 61-614 Poznan, Poland

## Abstract

We undertook the issue of the distribution of intracellular bacteria among Oribatida (Acari). Six genera of bacteria were detected by PCR and Sanger DNA sequencing: *Wolbachia*, *Cardinium*, *Rickettsia*, *Spiroplasma*, *Arsenophonus*, and *Hamiltonella*. Our research, for the first time, revealed the presence of *Cardinium* in *Microzetorchestes emeryi* in two subpopulations separated from each other by 300 m. The percentages of infected animals were the same in both subpopulations—ca. 20%. The identity of 16S rDNA sequences of *Cardinium* between these two subpopulations of *M. emeryi* was 97%. Phylogenetic analysis showed that the *Cardinium* in *M. emeryi* was clustered into the group A. The occurrence of *M. emeryi* in Poland has not been reported before and our report is the first one. *Cardinium* maybe help the thermophilic *M. emeryi* to adapt to low temperatures in the Central Europe.

## Introduction

Many bacterial species stay in close relationships with various invertebrate hosts, often establishing a persistent association. Symbiosis has an important role in the evolution of partners that are involved in the relation. The interplay of both organisms or, in some cases, between a host and more than one symbiotic bacterium, forms an evolving community that changes throughout time [1]. Sometimes, the relationship between host and bacterium is difficult to study, as some microorganisms are intracellular and cannot be cultured using standard bacteriological media under laboratory conditions [2]. Intracellular symbiosis is either obligate or facultative. Obligate endosymbionts are essential for hosts to survive and generally have long evolutionary histories with the organisms they live into [3]. Facultative endosymbionts can affect invertebrate’s development, reproduction [4], or protection from natural enemies [1, 5]. Endosymbiotic infection might even improve the pesticide resistance of hosts [6].

The oribatid mites (Oribatida: Acari) are one of the most abundant and common arthropod groups occurring in soil habitats [7]. However, the information of the endosymbiotic bacteria in these mites is poor. Intracellular microorganisms were found only in three oribatid species: *Oppiella nova* [8], *Hermannia gibba* [9], and *Achipteria coleoptrata* [10]. The role of the bacterial partners has not been discovered. It is especially interesting as these mite species are parthenogenetic [7] and some symbionts were found to be associated with parthenogenesis in arthropods [11–13]. Vertically transmitted bacteria *Wolbachia* [11], *Rickettsia* [12], and *Cardinium* [13] may change the way of reproduction of arthropods from sexual to parthenogenetic. Other maternally inherited bacteria are *Spiroplasma, Arsenophonus* [14], and *Hamiltonella* [15]. The richness of functions that intracellular bacteria can perform for their hosts is enormous. Some of them can eliminate males from the animal population also by male-killing, feminization, or cytoplasmic incompatibility. Some effects induced by bacteria do not affect reproduction or change in the number of males, but may affect nutritional balance [16]. The microorganisms may even protect invertebrates against viruses [17].

The intracellular bacteria of invertebrates can be detected by methods based on PCR and Sanger DNA sequencing [18–23]. Primers targeting the partial sequences of 16S rDNA have been established for detecting *Wolbachia* [18], *Cardinium* [19], *Rickettsia* [20], *Spiroplasma* [21], *Arsenophonus* [22], and *Hamiltonella* [23]. Sequencing of bacterial 16S rDNA is a universal tool for phylogenetic analysis of bacteria and molecular evolutionary research. It allows characterizing microorganisms in a diverse range of hosts and investigating relationships between bacteria. The analysis of 16S rDNA has discriminatory power and ability to classify strains into phylogenetic groups. An example is *Cardinium*. Based on 16S rRNA data, the genus has been divided into groups A, B, C [24], D [25, 26], and E [10].

We undertook the issue of the distribution maternally inherited bacteria among oribatid mites to fulfil the gap in the information about the occurrence of intracellular microorganisms in this group of mites. The endosymbionts were detected in mites isolated from soil samples of forest situated inside a town. We searched for *Wolbachia*, *Cardinium*, *Rickettsia*, *Spiroplasma*, *Arsenophonus*, and *Hamiltonella* by using PCR method. Phylogenetic analysis of *Cardinium* was based on 16S rRNA gene. A hypothesis “*M. emeryi* posses intracellular bacterial endosymbionts that were previously described in arthropods host species” was tested.

## Materials and Methods

### Collecting of Oribatida

Oribatid mites were collected from litter and soil samples (six samples from both localities to the depth of 10 cm) obtained in an urban mixed forest in the northern part of Poznań, Poland (52º27′N 16º56′E) of a temperate climate with relatively cold winters and warm summers. Tree species there were: pine (*Pinus sylvestris*), oak (*Quercus robur*), bird cherry (*Prunus padus*), and robinia (*Robinia pseudoacacia*). Samples were gathered from two mite localizations separated from each other by 300 m, representing similar type of vegetation and soil type, from May to July in years 2013–2016. Mites were extracted by using high-gradient Tullgren funnels, segregated intravitally and immediately placed directly in 96% ethanol. Comparative specimens were conserved in 70% ethanol and then, after clearing in lactic acid, identified to the species or genus level by using the Weigmann key [27]. Over 100 individuals belonging to the following families of oribatid mites were subjected to molecular testing: Hypochthoniidae, Nothridae, Camisiidae, Gymnodamaeidae, Damaeidae, Oppiidae, Carabodidae, Achipteriidae, Oribatulidae, Scheloribatidae, and Galumnidae.

### Screening of Bacterial Endosymbionts in Mites

The detection procedures of *Cardinium*, *Wolbachia*, *Arsenophonus*, *Spiroplasma*, and *Rickettsia* were described in detail previously [8]. DNA was extracted from a pool of 2–10 specimens by using Genomic Mini kit for universal genomic DNA isolation (A&A Biotechnology) according to the manufacturer’s instruction. Bacteria were detected using PCR. A reaction mixture contained 10–30 ng of DNA, 1x PCR buffer (Novazym), 0.2 mM dNTP (Novazym), 0.6 μM each primer (Oligo.pl), 0.4 U HiFi Taq polymerase (Novazym), and sterile bidistilled water to a total volume of 10 μl. Negative controls with no DNA were included in each reaction. Positive control of DNA isolation and PCR reaction was amplification of the mite 28S rRNA gene [28]. *Wolbachia* [29], *Cardinium* [30], and *Spiroplasma* [31] were detected by 16S rDNA sequencing. *Rickettsia* was detected by sequence analysis of the gene coding for *Rickettsia* 17 kDa protein antigen [32]. 23S rRNA gene was amplified for *Arsenophonus* detection [33]. The presence of *Hamiltonella* was determined by *Hamiltonella* chromosomal replication initiator protein [34]. PCR reactions were performed in a MyCycler thermal cycler (Bio-Rad). Amplicons were electrophoresed (Agarose NOVA Mini, Novazym) with a Nova 100 bp DNA Ladder (Novazym). Electropherograms were documented with the Bio-Print V.99 system (Vilber Lourmat). PCR products were directly sequenced or cloned by using TOPO TA Cloning Kit for Sequencing (Invitrogen) in accordance with the manufacturer’s instructions before sequencing. Sequencing were performed with BigDye Terminator v3.1 on an ABI Prism 3130XL (Applied Biosystems) and the sequences were compared to the available GenBank data (International Nucleotide Sequence Database Collaboration) by using BLASTn. The 326-bp 16S rDNA sequence of *Cardinium* in *M*. *emeryi* was deposited in GenBank under Accession No. KY039581.

### Phylogenetic Analysis of *Cardinium*

Phylogenetic analysis was performed based on 16S rDNA gene sequences. DNA was extracted from an individual specimen as described above. Because there is no noticeable external dimorphism in most oribatid mites (including *Microzetorchestes emeryi*), sex could be determined by examining the genitalia, which were usually retracted within the body. Unfortunately, this is not possible without lactic acid clearing of all specimens, which prevents later molecular procedures.

Detection of the gene was performed in 15 μl containing 10–30 ng of template DNA, 1x PCR DreamTaq Buffer (Thermo Scientific), 0.2 mM dNTP (Novazym), 0.75 μM each primer from one of the primer sets (Oligo.pl), and 0.4 U DreamTaq Hot Start DNA Polymerase (Thermo Scientific). The *Cardinium* 16S rDNA gene was amplified (1) with specific primer Ch-F and universal eubacterial primer 1513R [30], and (2) with the specific primer Ch-R and universal eubacterial primer 63F [35]. PCR products were analysed by electrophoresis, sequenced and compared with the available GenBank sequence data as described above. 16S rDNA sequences of *Cardinium* in *M*. *emeryi* from two subpopulations separated from each other by 300 m were deposited in GenBank under Accession No. MG889458 and MG889459.

The phylogenetic relationship of *Cardinium* from *M. emeryi* and other arthropod hosts was analysed using 45 16S rDNA sequences of *Cardinium* available in GenBank, including those detected in this study. Our aligned dataset consisted of 16S rDNA sequences of *Cardinium* symbionts from 28 insect hosts, 16 mite hosts including *M. emeryi*, one nematode, and one crustacean host. Sequences of *Amoebophilus asiaticus*, Bacteroidetes, and endosymbiont of *Acanthamoeba* sp. were used as an outgroup. The sequences were aligned with the use of CLUSTAL W with MEGA 6.06 software [36]. The appropriate model of sequence evolution was chosen using jModelTest 2.0 software [37]. The Hasegawa–Kishino–Yano model with C-distributed rates and invariant sites (G+I) was selected to compute genetic distances (base substitutions per site) [36]. The phylogenetic tree was constructed with the maximum-likelihood method and the bootstrap support was determined with 1000 bootstrap replicates.

## Results

This is the first report of *Cardinium* in *Microzetorchestes emeryi*. The bacterium was found in *M. emeryi* isolated from metapopulation. In the phylogenetic analysis using two pairs of primers Ch-F + 1513R and Ch-R + 63F, the bacterial 16S rDNA sequences with the length of 1443 bp and 1296 bp were obtained from mites of two subpopulations separated from each other by 300 m. The sequence of 1443 bp was obtained for *Cardinium* in *M. emeryi* from one subpopulation and the sequence of 1296 bp was obtained for *Cardinium* in *M. emeryi* from second subpopulation. These sequences were deposited in GenBank under Accession No. MG889458 and MG889459, respectively. This is the first report of this bacterium in *M. emeryi*. We examined 15 specimens of *M. emeryi* from each subpopulation. Three of them were infected in each subpopulation. Other bacterial symbionts (*Rickettsia*, *Spiroplasma*, *Arsenophonus*, and *Hamiltonella*) were not found in the examined mite individuals.

The G + C content of *Cardinium* 16S rDNA sequences was 49%. Within 16S rDNA, two sequences unique to the bacterium: 5′-GCGGTGTAAAATGAGCGTG-3′ and 5′-GGTCTTTAACTGACGCT-3′ [38] were found in *Cardinium* of *M. emeryi* in both subpopulations. The signature sequence for 16S rDNA of *Cardinium* 5′-GTATTTTGCTACTTTG-3′ designated by Zchori et al. [38] was not found in the sequence of the bacteria of *M. emeryi*. Instead, similar sequence 5′-GTATTTTGCCACCTTG-3′ was found in *Cardinium* 16S rDNA of *M. emeryi* from both subpopulations.

The comparison of 16S rDNA sequences of *Cardinium* in *M. emeryi* with those in other arthropod hosts, deposited in GenBank, by using BLASTn revealed that the sequence of *Cardinium* in *M. emeryi* of one subpopulation (Accession No. MG889458) was most similar to sequences of Bateroidetes endosymbiont of mite *Metaseiulus occidentalis* (Accession No. AY753170 and AY753169) with 98% identity and 0.0 E value. Within 16S rDNA of *Cardinium* of *M. occidentalis*, the first sequence of the unique to the bacterium was found. The second unique sequence and the signature sequence differed in one nucleotide and were as follows: 5′-GGTCTTTGACTGACGCT-3′ and 5′-GTATTTTGCTACCTTG-3′, respectively. The 16S rDNA of *Cardinium* in *M. emeryi* of the second subpopulation (Accession No. MG889459) showed highest similarity to the uncultured bacterium of mite *Dermatophagoides farinae* (Accession No. JN236339), Bacteroidetes endosymbiont of mite *Metaseiulus occidentalis* (Accession No. AY753170), and *Cardinium* of insect *Bemisia tabaci* (Accession No. LC159289) with 97% identity and 0.0 E value. Within 16S rDNA of *Cardinium* of *D. farinae* and *B. tabaci* two unique sequences to the bacterium were found. The signature sequence of *Cardinium* in *D. farinae* differed in one nucleotide and it was 5′-GTGTTTTGCTACCTTG-3′. The signature sequence of the bacterium from *B. tabaci* was identical to that found in *Cardinium* from *M. emeryi*. The similarity of sequences of *Cardinium* in. *M. emeryi* of both subpopulations was also high with identity 97% and 0.0 E value. We also compared the sequences of *Cardinium* 16S rDNA of *M. emeryi* of both subpopulations, *Oppiella nova* (Accession No. AY279414), and *Achipteria coleoptrata* (Accession No. MG889457) as they are *Cardinium* hosts representing Oribatida. The similarities of sequences were high with identity 93–98%. Within 16S rDNA of *Cardinium* of *O. nova*, the first unique sequence to the bacterium was not found, whereas the second one was noted. The signature sequence in *Cardinium* from *O. nova* differed in two nucleotides and it was 5′-GTGTTTTGCTACCTTG-3′. The first of the unique sequence was noted in *Cardinium* from *A. coleoptrata*. In the second unique sequence, Konecka & Olszanowski [10] found one nucleotide substitution in the 7th position (A instead of T) and this sequence was 5′-GGTCTTAAACTGACGCT-3′.

**Fig. 1:**
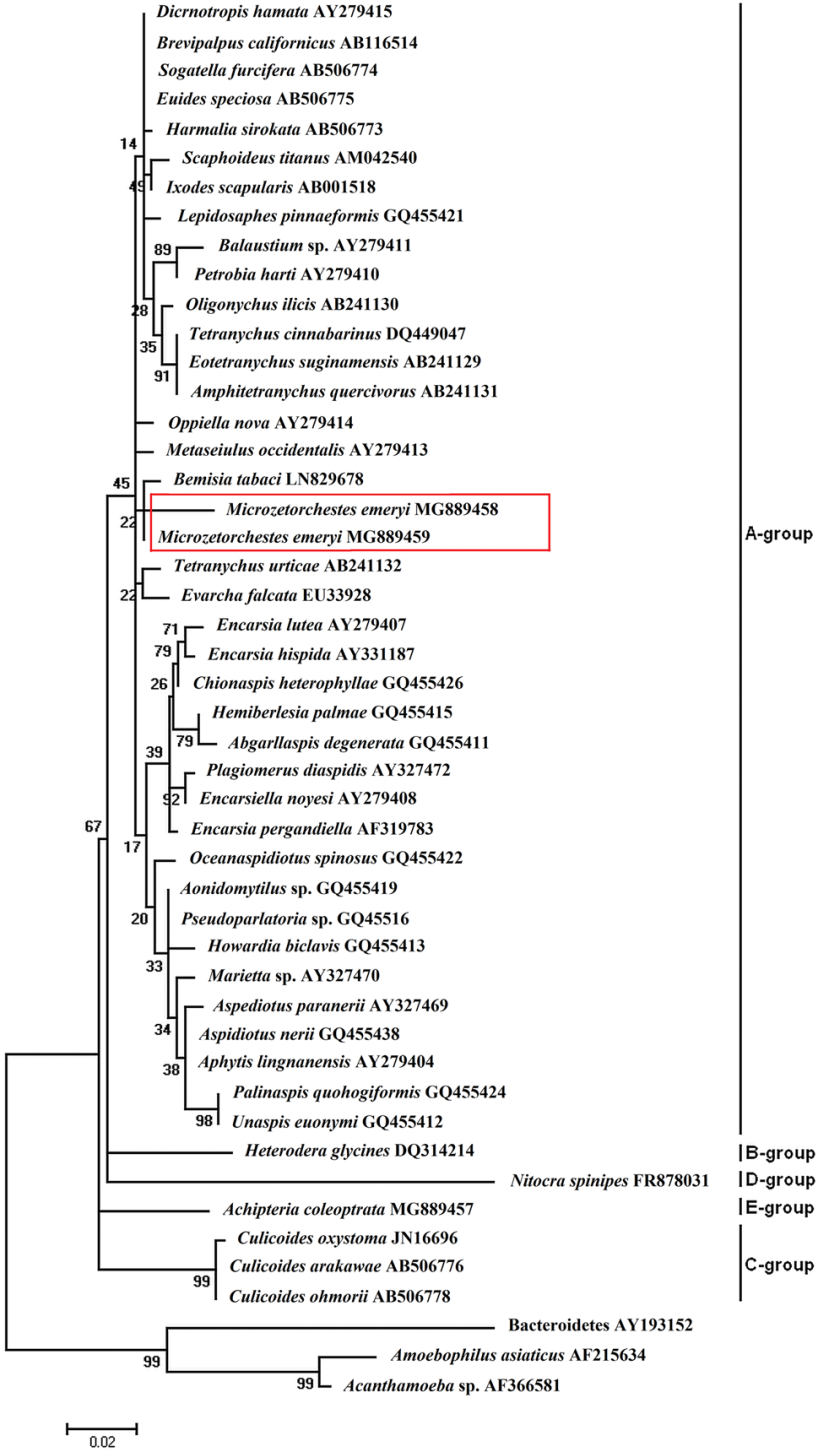
Phylogeny based on the sequences of *Cardinium* 16S rDNA gene. Strains of *Cardinium* were designated by the names of their host. *Amoebophilus asiaticus*, Bacteroidetes, and endosymbiont from *Acanthamoeba* sp. were used as an outgroup. Bar, substitutions per nucleotide. Bootstrap values based on 1000 replicates are shown on the branches

The phylogenetic analysis revealed *Cardinium* of *M. emeryi* belonged to group A with the nearest branch of *Cardinium* in *B. tabaci* (Fig. 1). *Cardinium* found in other oribatid host—*O. nova* was also clustered within group A, together with *Cardinium* in *M. emeryi*.

## Discussion

The study concerns distribution of microorganisms living inside Oribatida mites. Bacteria *Cardinium* were detected, for the first time, in oribatid mites *Microzetorchestes emeryi* from two subpopulations of metapopulation. The percentages of infected animals were the same in both subpopulation—ca. 20%. Similar rate of bacterial occurrence was observed in other arthropod hosts [24, 39]. However, in some invertebrate populations, the frequency of *Cardinium* prevalence was higher [24, 40]. Finding uninfected individuals indicated that the bacteria do not function as primary symbionts. The G-C content (49%) was the same in 16S rDNA of *Cardinium* from *M. emeryi* of both subpopulations and in *Cardinium* previously found in other arthropods [24]. The bacterial strains infecting *M. emeryi* from neighbouring places were similar based on 16S rDNA sequences to bacteria found in mite *Metaseiulus occidentalis* and insect *Bemisia tabaci*. The sequences of *Cardinium* 16S rDNA in mites *Oppiella nova* and *Achipteria coleoptrata* also showed high level of similarity to *Cardinium* in *M. emeryi* representing the same order Oribatida.

Although the sequences of 16S rDNA of *Cardinium* strains from *M. emeryi* of neighbouring subpopulations differed, they showed high level of similarity 97%. Phylogenetic analysis revealed that the bacteria were closely related and formed sister branches together with *Cardinium* of *B. tabaci* (Fig. 1). No data, except from 16S rDNA sequence deposited in GenBank, are available on the *Cardinium* strain in *B. tabaci* from the sister branch of *Cardinium* in *M. emeryi* and no information was given on its impact on its insect host. The role of the endosymbiont in the insect is unclear and has not been discovered. However, other *Cardinium* strains were not proven to be feminization, cytoplasmic incompatibility, and parthenogenesis inductors in *B. tabaci* [41], but their function in the insect male-killing was suspected [23]. Some literature data suggest that *Cardinium* may be a mutualistic symbiont [42], whereas other data indicate opposite symbiotic relation [41]. The bacterial beneficial abilities for the insect host probably are related to *Cardinium* motility and toxin synthesis. The genome of the symbiont of *B. tabaci* includes gliding genes that are related to bacterial motility and also the putative toxin-related genes encoding proteins related with bacterial insecticidal toxins. Due to the motility feature, *Cardinium* may directly contact a host’s parasitoid and kill it by secreting toxins [26]. Other literature data revealed negative role of *Cardinium* in *B. tabaci*. The *Cardinium*-free insects had higher fitness than *Cardinium*-infected *B. tabaci* [41]. It cannot be excluded that the action of bacteria may depend on *Cardinium* strain, its properties, and/or the *B. tabaci* population and the characteristic features of insects belonging to the population. In *M. emeryi* metapopulation, the *Cardinium* role of inducing parthenogenesis, feminization, and male-killing could be rejected as *M. emeryi* is a sexual species [43]. *M*. *emeryi* is a thermo-xerophilous species [44] and has a more southern distribution in Europe [27, 45]. There is no literature data on the occurrence of this mite in Poland. We detected, for the first time, two neighbouring localizations of *M. emeryi* in an urban, mixed forest in the north-western part of Poland. Additionally, in both subpopulations infected mites were found. The occurrence of *M. emeryi* in Poland is enigmatic. The Polish climate is mostly temperate. The winter temperatures dropped to around − 6 °C in the last few years and with average rainfall during few last years of ca. 600 mm (data from the website of The Institute of Meteorology and Water Management—National Research Institute). Although the average temperatures are rising [46], it is still difficult to explain why *M. emeryi* mites that prefer rather warm and dry climate of Mediterranean regions were observed in Central Europe. The phenomenon of global warming resulting in the emergence of southern species in Polish fauna and flora could be also important. Perhaps *M. emeryi* is a relatively recent component of the local fauna. Did *Cardinium* have an impact on the survival of their hosts at low temperatures? It has been shown that temperature change may influence the community of symbiotic bacteria in invertebrates. The dominant bacterial species in different generations of insects are stable under low temperatures, but can significantly change under high temperatures. High temperature reduces the number of some symbiotic bacterial species in insects [47]. Possibly, the microbes allow surviving invertebrates at low temperatures and this is why the changes in the species composition of the host’s microbiome due to temperature changes were observed. *Cardinium* maybe help the *M. emeryi* mites to adapt to low temperatures in the Central Europe. It seems interesting that *B. tabaci* originated from tropical and subtropical regions and additionally one of the *B. tabaci* geographically defined clade is Mediterranean [48]. Now the insect is widely distributed in temperate areas including most of Europe [49]. Although there is no evidence for endosymbiont contribution in insect adaptation to colder and harsher climate, the history of *M. emeryi* and *B. tabaci* moving from dry summer to tepid regions is similar and the scenario of microbiome helpful role in their spreading cannot be excluded.

In conclusion, our research, for the first time, revealed the presence of *Cardinium* in oribatid mites *M. emeryi* in two subpopulations of metapopulation. Phylogenetic analysis showed that the bacteria clustered with *Cardinium* of other invertebrate hosts in group A. They were closely related and formed sister branches together with *Cardinium* of *B. tabaci*. The role of the endosymbiont in the mites is unclear. Possibly, the bacterium is the key to explaining the mystery of the occurrence of these thermophilic mites in the colder climate of Central Europe.
